# An age-old problem or an old-age problem? A UK survey of attitudes, historical use and recommendations by healthcare professionals to use healthcare apps

**DOI:** 10.1186/s12877-023-03772-x

**Published:** 2023-02-24

**Authors:** Sulayman Z. Chowdhury, Sebastian Stevens, Charlotte Wu, Claire Woodward, Tim Andrews, Liz Ashall-Payne, Simon Leigh

**Affiliations:** 1 The Organisation for the Review of Care and Health Applications (ORCHA), Sci-Tech Daresbury, Vanguard House, Keckwick Lane, Daresbury, WA4 4AB UK; 2 Warwick Medical School, University of Warwick, Warwick, UK

**Keywords:** Digital health, mHealth, Equity, Geriatric, Old-age, Equality, Ageing, Smartphone applications, Healthcare apps

## Abstract

**Background:**

The coronavirus pandemic has exacerbated barriers to accessing face-to-face care. Consequently, the potential for digital health technologies (DHTs) to address unmet needs has gained traction. DHTs may circumvent several barriers to healthy independent living, resulting in both socioeconomic and clinical benefits. However, previous studies have demonstrated these benefits may be disproportionately realised among younger populations while excluding older people.

**Methods:**

We performed a prospective survey using the One Poll market research platform among 2000 adults from the United Kingdom. To mitigate against self-selection bias, participants were not informed of the topic of the survey until they had completed recruitment. We compared willingness to use and historical use of health-apps, in addition to recommendations to use health-apps from healthcare professionals; comparing outcomes across all age groups, including a reference group (*n* = 222) of those aged 18-24. Outcomes were analysed using multivariate logistic regression and reported as odds ratios (OR) with respondent age, ethnicity, gender, and location as covariates.

**Results:**

Willingness to use health-apps decreased significantly with age, reaching a minimum (OR = 0.39) among those aged 65 and over compared to the reference group of 18-24 year olds. Despite this, more than 52% of those aged 65 and over were willing to use health-apps. Functions and features most cited as useful by older populations included symptom self-monitoring and surgery recovery assistance. The likelihood of never having used a health-app also increased consistently with age, reaching a maximum among those aged 65 and over (OR = 18.3). Finally, the likelihood of being recommended health-apps by a healthcare professional decreased significantly with age, (OR = 0.09) for those aged 65 and over. In absolute terms, 33.8% of those aged 18-24, and 3.9% of those aged 65 and over were recommended health-apps by their healthcare professionals.

**Conclusion:**

Although absolute utilisation of health-apps decreases with age, the findings of this study suggest that the gap between those willing to use health-apps, and those being recommended health-apps by healthcare professionals increases with age. Given the increasing availability of evidence-based health-apps designed for older populations, this may result in entirely avoidable unmet needs, suggesting that more should be done by healthcare professionals to recommend health-apps to older persons who are generally positive about their use. This may result in considerable improvements in healthy and independent ageing.

## Background

While the morbidity and mortality impact of the coronavirus (COVID-19) pandemic has been catastrophic [1], the resulting restrictions on face-to-face contact may have inadvertently encouraged the beginnings of a digital health paradigm shift. Despite a suggested appetite for digital health technologies (DHTs), which include health-apps, among patients [2], healthcare professionals [3, 4] and policymakers alike [5] prior to the COVID-19 pandemic, global engagement with DHTs has been minimal [6–9]. However, as we now attempt to balance our need to access healthcare against not only the desire to protect one another from transmission of COVID-19, but also increasing elective care backlogs [10], recent data suggest that interest in health-apps, has surged. One study analysing ~ 126,000 internet searches for health-apps in the UK over a two-year period demonstrated a 343% increase in internet searches for health-apps following the first COVID-19 lockdown in March 2020 [2].

While we find increasing availability of evidence to suggest health-apps may provide added value to older populations, including a recent systematic literature review of 344 studies [11], and a growing number of evidence-based solutions including NeuroNation [12], InspireD [13], Wysa [14], Fibricheck [15] and Cognifit [16] amongst others, numerous prior studies, conducted on a global scale, have shown that the benefits of digital health are more often disproportionately realised among younger populations [17, 18].

Another recent mixed-methods study involving 222 healthcare professionals from the UK, found that age was the single largest barrier in recommending evidence-based health-apps to patients. In this study, healthcare professionals were more likely to recommend a health-app with no published evidence, which cost the national health service (NHS) £15, to an 18-year-old patient, than to offer a health-app with a Randomised Controlled Trial (RCT), which was free to the NHS, to a 65-year old [17].

In this prospective study utilising a novel blinded survey approach, we analyse data from a large representative survey of 2000 individuals from the UK regarding their preferences and experiences with health-apps. Specifically, we explore willingness to use health-apps and contrast this with actual use, and whether respondents have historically been recommended health-apps by their healthcare professionals. Ultimately, this study aims to determine whether any unmet needs for digital inclusion exist, whether age plays a role, and what may be done to ensure the fair and inclusive use of clinically beneficial health-apps across healthcare systems.

## Methods

### Participants

Participants were adults (aged ≥18 years) residing in the United Kingdom and pre-registered with a large multinational market research agency, described in greater detail below. Participants were self-selected to complete our survey, while blinded to the title, theme and details of the survey, mitigating against the self-selection of highly digitally literate individuals with a specific interest in digital health.

### Eligibility

Prospective respondents were only required to be able to read and write in English. There were no other exclusion criteria applied except for location, with all respondents residing in the United Kingdom.

### Procedures

The cross-sectional survey was developed in line with the Checklist for Reporting Results of Internet E-Surveys (CHERRIES) reporting standards. The survey examined attitudes towards the perceived usefulness and historical use of health-apps. The survey also explored how respondents identified health-apps, including recommendations by their healthcare professionals, and their beliefs regarding the role of health-apps within existing National Health Service (NHS) care pathways. Prior to being offered to participants the survey was tested internally by two members of the research team (SL and SZC) and piloted among a small group of respondents to assess usability, readability and interpretation. Recruitment for the survey, described below, was conducted over three working days in June 2021. The survey was administered through One Poll, an international market research agency specializing in online and mobile polling, with 70,000 members of which to distribute surveys.

Previous research has found that crowdsourcing platforms like One Poll allow for rapid and inexpensive capture of high-quality survey data from a large and potentially more diverse population than typically seen in standard convenience samples. We aimed for a large sample of 2000 to capture a representative view of UK adults and enable subgroup analyses with sufficient power, informed where possible, by evidence demonstrating their impact on utilisation of digital health, including age [22], location, gender and ethnicity [23].

We used an open quota survey approach, with the survey advertised to each registered One Poll user residing in the UK and remaining available until the required sample of participants was reached. The survey was offered in English language only using a web form. Participants were informed approximately how long the survey would take, but not told anything about the aims, objectives or theme of the survey before taking part, resulting in a novel “blinded survey” to mitigate against self-selection by those with a specific interest in digital health, and to protect against de-selection by those with no interest in or experience of digital health. No personally identifiable data were collected or stored, with all participants reimbursed for their participation in the survey. Given the short duration required to complete the survey (approximately 5 min), the order in which questions appeared was not randomised as we anticipated the likelihood of survey fatigue to be minimal in such a short survey. All questions were provided on separate pages.

Data integrity checks and mechanisms to protect against unauthorized access were built into the study design, including ensuring unique visitors by requiring participants to register their IP when completing the survey. IP checks remained in place throughout the survey, protecting against participants repeatedly answering the survey for additional financial rewards. Additionally, participants could not amend, review or change their answers to a question once submitted, with no back button and no summary page of responses provided.

### Measures

**Fig. 1:**
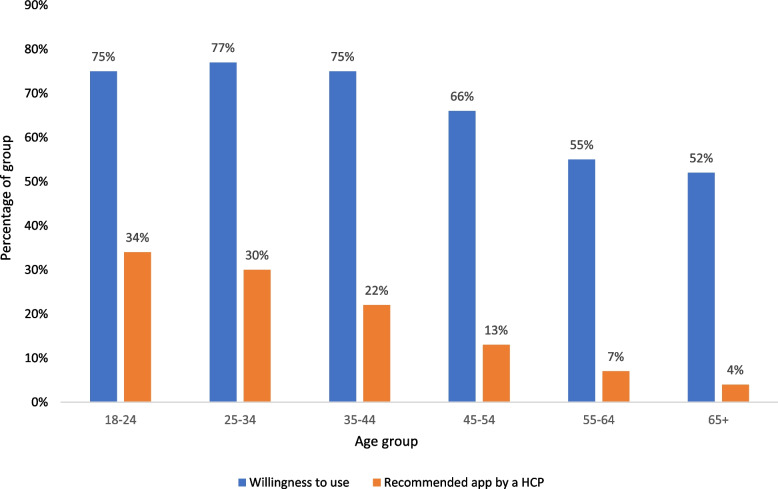
Willingness to use, & HCP recommendations to use health-apps by age group

Survey questions were developed by the principal investigator. The survey was comprised of structured questions with no free text options provided. Participants were asked the following sequentially answered questions:

1. Tick the statements you agree with/apply to you. ‘I’d be willing to use a top-quality, clinically backed health app to…’ [multiple answer, various use cases shown in Table 1]
2. To what extent do you agree or disagree with this statement? ‘In order to help the NHS, it is vital we all look at new ways to manage our health, including using high-quality health apps, not just during the pandemic but into the future.’ [multiple choice, 5-point Likert scale, responses shown in Fig. 1]
3. To what extent do you agree or disagree with this statement?’ If it ultimately saves the NHS money, doctors should be able to prescribe high-quality health apps which charge for their services, in the same way they prescribe traditional medicines.’ [multiple choice, 5-point Likert scale, responses shown in Fig. 1]
4. If you have ever used a health app to help with your physical or mental health, please tell us who recommended the app to you? [multiple answer, answers then summarised to be HCP or non HCP and used in logistic regression in Table 6]

**Table 1:**
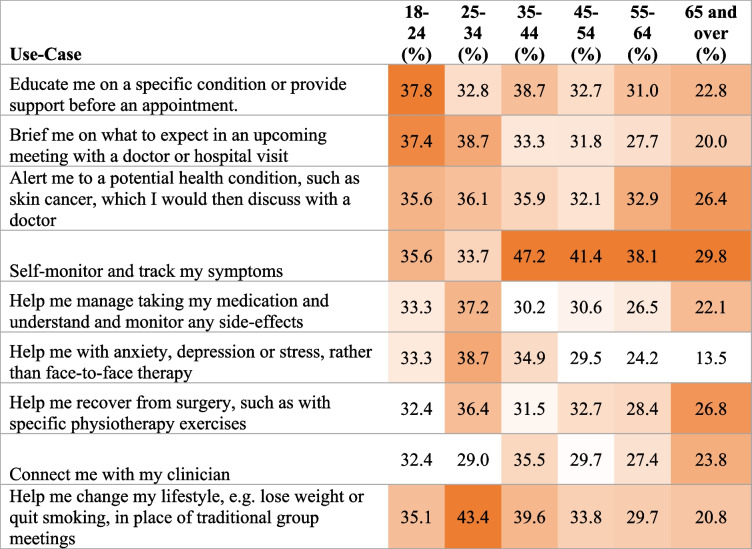
Heat Map showing % of age group agreement with listed use-cases of health-apps

Heat map uses 2-colour scale from white (lowest value within an age group) to orange (highest value of that age group)

We additionally collected demographic characteristics of respondents including location, gender, age and ethnicity. Because of small sample sizes across minority groups, racial and ethnic identity was collapsed to create a binary variable of white, or black and minority ethnic group (BAME). Each multiple-choice question was scored on a five-point Likert scale (strongly disagree, disagree, neutral, agree, strongly agree).

### Statistical analysis

We imported participant responses into Microsoft® Excel™ (Redmond, WA). We present summary statistics to describe the characteristics of the participants. Categorical variables were summarised by frequency and percentage, with continuous variables reported as mean and standard deviation (SD). We used multivariate logistic regression to calculate odds ratios with robust standard errors, adjusted for age, sex, ethnicity and location. We use the youngest age group (18-24) as the reference group for chronological consistency and easier interpretation of results. This group is also shown to be the most digitally active in previous studies [22]. All statistical analyses were conducted using STATA 14 (StataCorp LP, USA) with statistical significance defined at the conventional 5% level.

### Data availability

Those wishing to utilise the study data for non-commercial purposes can request access to the data by contacting the corresponding author.

### Ethics

We We received ethical approval from the Biomedical and Scientific Research Ethics Committee (BSREC) at the University of Warwick Research Governance and Ethics Committee, under agreement number 64/22-23. Additionally, we received explicit informed consent from the data controller and individual data providers to access and utilize the data collected specifically for market research.

## Results

### Baseline characteristics

Two-thousand and fifty-one adults responded to the survey during the study period, with 51 failing to complete the survey, resulting in 2000 responses and a 97.5% completion rate. In terms of age, the largest group constituted individuals aged 65 and over (23.3%), followed by those aged 45-54 (17.2%) as shown in Table 2. Females accounted for 51.5% of our sample, and males 48.5%. Most respondents resided in the Southeast (14.1%) or London (13.6%). Finally, ~ 85% of the sample identified as white British, which although high, corresponds to the approximate underlying proportion of white British individuals within the United Kingdom. Further details regarding the study sample are provided in Table 2.

**Table 2:** Baseline characteristics of UK survey sample

Parameter	Item	Frequency	% of Sample (***n*** = 2000)
**Age**	18-24	222	11.10
	25-34	341	17.05
	35-44	318	15.90
	45-54	343	17.15
	55-64	310	15.50
	65 and Over	466	23.30
**Sex**	Male	970	48.50
	Female	1030	51.50
**Location**	East Anglia	185	9.25
	East Midlands	147	7.35
	London	272	13.60
	Northeast	80	4
	Northern Ireland	56	2.80
	Northwest	219	10.95
	Scotland	165	8.25
	Southeast	282	14.10
	Southwest	172	8.60
	Wales	90	4.50
	West Midlands	168	8.40
	Yorkshire and the Humber	164	8.20
**Ethnicity**	Arab or Arab British	1	0.05
	Asian or Asian British – Bangladeshi	5	0.25
	Asian or Asian British – Chinese	18	0.90
	Asian or Asian British – Indian	30	1.50
	Asian or Asian British – Other	9	0.45
	Asian or Asian British – Pakistani	16	0.80
	Black or Black British – African	46	2.30
	Black or Black British – Caribbean	37	1.85
	Black or Black British – Other	1	0.05
	Mixed / Multiple – Other	3	0.15
	Mixed / Multiple – White and Asian	13	0.65
	Mixed / Multiple – White and Black African	4	0.20
	Mixed / Multiple – White and Black Caribbean	7	0.35
	Other ethnicity	11	0.55
	White – British	1702	85.10
	White – Gypsy / Traveller / Irish traveller	6	0.30
	White – Irish	38	1.90
	White – Other	53	2.65

### The value of functions & features

The heat map in Table 1 demonstrates willingness to use health-apps with varying functions and features by age group (relating to question 1 in the survey). While younger individuals are most likely to value and utilise health-apps with all the use-cases listed, older individuals valued some functions and features more than others. Only 13.5% of those aged 65 and over reported being willing to use mental health services delivered digitally rather than face-to-face.

While conversely, “alerting me to a potential health condition, such as skin cancer, which I would then discuss with a doctor” (26.4%), “self-monitoring and tracking my symptoms” (29.8%), and “helping me recover from surgery, such as with specific physiotherapy exercises” (26.8%) were considered of greater value to older persons with self-monitoring being the most prevalent desired use-case amongst all older age groups 35 and above.

### Willingness to use health-apps

Table 3 demonstrates the relationship between respondent characteristics and willingness to use or be prescribed a health-app. Willingness to use health-apps decreased consistently with age, reaching statistical significance versus the comparator group (those aged 18-24) among those aged 45 and over, and reaching a minimum among those aged 65 and over, where the willingness to use health-apps reduced by 61% [95% CI 43-73%], compared to the reference group. Additionally, with the exception of the Southeast, respondents from all locations were significantly less likely to be willing to use health-apps than those from London.

**Table 3:** Logistic regression demonstrating willingness to utilise health-apps

Variable	Odds Ratio	95% Confidence Interval
18-24 (Reference Age Group)		
25-34	1.07	[0.72;1.60]
35-44	0.98	[0.66;1.46]
45-54	0.68^*^	[0.47;0.99]
55-64	0.42^*^	[0.29;0.62]
65 and over	0.39^*^	[0.27;0.57]
Male (vs. Female)	0.90	[0.74;1.09]
BAME (vs. White British)	0.88	[0.61;1.26]
London (Reference Location)		
East Anglia	0.52^*^	[0.34;0.81]
East Midlands	0.54^*^	[0.34;0.86]
Northeast	0.44^*^	[0.25;0.78]
Northern Ireland	0.29^*^	[0.15;0.55]
Northwest	0.41^*^	[0.27;0.62]
Scotland	0.62^*^	[0.39;0.97]
Southeast	0.72	[0.48;1.08]
Southwest	0.53^*^	[0.34;0.83]
Wales	0.44^*^	[0.26;0.75]
West Midlands	0.50^*^	[0.32;0.78]
Yorkshire and the Humber	0.65^*^	[0.41;1.03]
Constant	5.39^*^	[3.48;8.35]

^*^Denotes statistical significance at 5% level (*p* < 0.05)

### Beliefs concerning NHS funding of paid health-apps

Table 4 demonstrates the relationship between respondent characteristics and agreement with the NHS funding of paid health-apps. For most older age groups, there was a significant decrease in agreement with NHS funding as the age group increases, between 18 and 24 to 25-34 there is a 47% drop in the odds of agreeing to apps charging the NHS, this drops further until, for 65 and over, there is an 85% decrease in odds showing a strong linear downwards trend with regards to age at high statistical significance for nearly all age groups. Further mean analysis shows that around 73% of 18–24 year-olds agree with the NHS paying for apps, while only 58% agree in the next age group, this falls to 26% in our oldest group.

**Table 4:** Logistic regression demonstrating agreement for NHS funding of paid health-apps

Variable	Odds Ratio	95% Confidence Interval
18-24 (Reference Age Group)		
25-34	0.53^*^	[0.37;0.77]
35-44	0.71^*^	[0.49;1.04]
45-54	0.39^*^	[0.27;0.56]
55-64	0.28^*^	[0.19;0.41]
65 and over	0.15^*^	[0.11;0.22]
Male (vs. Female)	1.31^*^	[1.09;1.58]
BAME (vs. British White)	1.20	[0.86;1.66]
London (Reference Location)		
East Anglia	0.54^*^	[0.36;0.81]
East Midlands	0.45^*^	[0.29;0.70]
Northeast	0.78	[0.45;1.35]
Northern Ireland	0.47^*^	[0.24;0.90]
Northwest	0.50^*^	[0.34;0.73]
Scotland	0.52^*^	[0.34;0.79]
Southeast	0.77	[0.54;1.10]
Southwest	0.69	[0.46;1.03]
Wales	0.64	[0.38;1.07]
West Midlands	0.58^*^	[0.38;0.88]
Yorkshire and the Humber	0.67	[0.44;1.02]
Constant	3.38^*^	[2.28;5.01]

^*^Denotes statistical significance at 5% level (*p* < 0.05)

### Historical use of health-apps

Table 5 explores the factors associated with prior use of health-apps by respondents. As age increased the likelihood of having never used a health-app increased consistently, with all age groups significantly less likely to have used health-apps than those aged 18-24, reaching a maximum 18.3-fold reduction [95% CI 12.0-27.9-fold] among those aged 65 and over. Similarly, respondents located in all regions outside of London were significantly less likely to have used a health-app than those residing in London. Respondents who were male or part of the BAME community were significantly more likely to have used a healthcare app.

**Table 5:** Logistic regression demonstrating the likelihood of having never used a health-app

Variable	Odds Ratio	95% Confidence Interval
18-24 (Reference Age Group)		
25-34	1.69^*^	[1.14;2.49]
35-44	2.73^*^	[1.86;4.02]
45-54	6.28^*^	[4.27;9.25]
55-64	10.95^*^	[7.13;16.82]
65 and over	18.27^*^	[11.97;27.89]
Male (vs. Female)	0.68^*^	[0.55;0.84]
BAME (vs. British White)	0.67^*^	[0.47;0.96]
London (Reference Location)		
East Anglia	4.20^*^	[2.67;6.62]
East Midlands	4.47^*^	[2.71;7.36]
Northeast	2.34^*^	[1.26;4.35]
Northern Ireland	4.14^*^	[1.99;8.61]
Northwest	2.57^*^	[1.65;4.00]
Scotland	4.30^*^	[2.71;6.83]
Southeast	1.84^*^	[1.26;2.68]
Southwest	3.16^*^	[1.99;5.00]
Wales	2.85^*^	[1.63;4.99]
West Midlands	3.24^*^	[2.06;5.08]
Yorkshire and the Humber	3.15^*^	[2.01;4.94]
Constant	0.18^*^	[0.12;0.27]

^*^Denotes statistical significance at 5% level (*p* < 0.05)

### Recommendation to use health-apps by healthcare professionals

Table 6 demonstrates the relationship between respondent attributes and whether they have previously been recommended or prescribed a health-app by a healthcare professional. Every age group over the age of 35 was significantly less likely to be recommended health-apps by healthcare professionals than those aged 18-24 (the reference group), reaching a minimum among those aged 65 and over, who were 91% less likely to be recommended a health-app than those aged 18-24. In absolute terms 33% of those aged 18-34 were recommended health-apps by a healthcare professional, reducing to 21% among those aged 35-44, 13% for those aged 45-54, 7% for those aged 55-64 and 4% for those aged 65 and over. Also, male or being part of BAME respondents were statistically more likely to have been recommended a health-app by a healthcare professional. Additionally, respondents from all locations included were statistically significantly less likely to be recommended health-apps by healthcare professionals than those residing in London.

**Table 6:** Logistic regression demonstrating the likelihood of being recommended a health-app by a HCP

Variable	Odds Ratio	95% Confidence Interval
18-24 (Reference Age Group)		
25-34	0.75	[0.51;1.11]
35-44	0.54^*^	[0.36;0.81]
45-54	0.32^*^	[0.21;0.49]
55-64	0.17^*^	[0.10;0.29]
65 and over	0.09^*^	[0.05;0.16]
Male (vs. Female)	1.48^*^	[1.15;1.92]
BAME (vs. British White)	1.51^*^	[1.04;2.19]
London (Reference Location)		
East Anglia	0.34^*^	[0.20;0.57]
East Midlands	0.34^*^	[0.19;0.61]
Northeast	0.28^*^	[0.13;0.63]
Northern Ireland	0.14^*^	[0.05;0.40]
Northwest	0.45^*^	[0.27;0.73]
Scotland	0.26^*^	[0.15;0.45]
Southeast	0.35^*^	[0.23;0.54]
Southwest	0.35^*^	[0.20;0.60]
Wales	0.53^*^	[0.29;1.00]
West Midlands	0.39^*^	[0.23;0.65]
Yorkshire and the Humber	0.30^*^	[0.17;0.54]
Constant	0.92	[0.60;1.41]

^*^Denotes statistical significance at 5% level (*p* < 0.05)

### Disparity between willingness to use health-apps and recommendation to use health-apps by healthcare professionals

Figure 1 highlights the disparity between people’s willingness to be prescribed a health-app and the likelihood of being prescribed health-apps by a healthcare professional. Among those willing to use a health-app, the proportion prescribed health-apps by healthcare professionals, which may act as a proxy for unmet needs in accessing healthcare services, decreases consistently with age. Approximately 45.3% of those willing to use health-apps are currently recommended them among those aged 18-24, falling to 29.3% (35-44), 19.7% (45-54), 12.7% (55-64) and 7.7% among those aged 65 and over (Fig. 1).

## Discussion

### Principal findings

This study, which utilises a novel approach of blinding study respondents to the subject of the survey prior to participation, has highlighted that age is a significant determinant of perceived value, utilisation rates, healthcare professional recommendations and unmet needs in accessing health-apps. While it was demonstrated that interest in using health-apps, and beliefs that the NHS should fund access to health-apps decreased with age, we highlight that more than 50% of respondents aged 65 and over would still value the opportunity to use health-apps. Despite this, the odds of never having used a health-app were 18.3-fold higher among those aged 65 and over than in our reference group, those aged 18 to 24, suggesting that age is a key predictor of engagement with health-apps. Additionally, we show that this age-dependent increasing likelihood of having never used a health-app was linked to a lack of recommendations from healthcare professionals as patients age. Compared to those aged 18 to 24, the odds of being recommended a health-app by a healthcare professional reduced by 46% for 35-44 year-olds, 68% for those aged 45-54, 83% for those aged 55-64 and 91% for individuals aged over 65. Taken together, the findings of this study demonstrate that the disparity between willingness to use a health-app, and recommendations to use health-apps by healthcare professionals, increases with age, thus exacerbating unmet needs in later years when need for high-quality healthcare is at its greatest.

We found that the utilisation of health-apps is both negatively and consistently associated with age, with those aged 55-64 and 65 and older, 10.9 and 18.3-times less likely to have used a health-app than those aged 18-24. This finding may be explained by several factors. Recent research has demonstrated that approximately 25% of the UK general population suffers from multi-morbidity [24], with this figure increasing to the extent that the National Institute for Health and Care Excellence (NICE) have recently published guidance on the management of those with multiple LTCs [25]. Currently, most health-apps focus on specific condition areas, and as such, for individuals with multiple conditions, this may require the use of several health-apps simultaneously, thereby increasing barriers and reducing incentives for use. Technological advances including health-apps which cater not to specific conditions but to holistic symptom management, thereby incorporating concerns across multiple condition areas, may increase rates of effective utilisation by negating the need for older persons to seek out multiple health-apps.

An alternative explanation for the lower rates of digital engagement found among older persons in this study concerns digital inclusivity and specifically, non-inclusive design. A recent systematic review conducted in 2021 highlighted that the effect of co-designed technology on health and well-being was rarely studied among older adults [21], while another review identified 42 different barriers to digital adoption which specifically affect older adults suffering from Alzheimer’s disease [26]. It is clear that with age-related decline, user needs concerning the accessibility of technologies will change, and with most digital health technologies designed with the ‘average’ user in mind, this may not be enough to ensure such technologies are accessible and usable for older persons. Future research and co-design efforts are therefore essential to highlight and address barriers unique to older adults, in order to maximise the potential for value to be derived from these technologies as highlighted by this recent systematic literature review [27].

This theme concerning the lack of user-centred design among older populations may also explain another of our findings, that all things being equal, healthcare professionals recommended health-apps to older persons significantly less than younger patients. We found that compared to those aged 18-24, those aged 45-54, 55-64 and 65+ were 68, 83 and 91% less likely to be recommended health-apps by a healthcare professional. With an ageing population, greater patient expectations of healthcare than ever before, and an increasing struggle to train and recruit clinicians, pressures on healthcare providers are greater than ever. As a group who are likely to require considerably greater utilisation of NHS services, this may explain why we observed a statistically significant decrease in agreement with NHS funding access of health-apps, as age increases. Furthermore, If healthcare professionals perceive that older persons may disproportionately have less knowledge and understanding of digital technologies, and therefore perceive that older persons are likely either to return to the clinic with problems or to require support in using health-apps, then this may explain lower rates of recommendation among this age group.

Healthcare professionals may therefore require additional support in recommending health-apps to older persons in a way which does not increase existing workload, including multidisciplinary support or more informed discussions with patients regarding their needs, this would help with the most common barriers of digital health with older peoples such as lack of support, knowledge and self-efficacy as previously demonstrated [28]. Shared decision making which details the implications and benefits derived from the digital intervention and how it may address the patient’s goals is likely to be essential in this process [26]. We provide a preliminary assessment of the value of several functions and features to older persons, but future research should aim to explore which features are most valued by persons of different ages, and with different conditions or symptom profiles, this, in turn, may accelerate the effective utilisation of health-apps among these groups.

Considerations around healthcare professionals’ perceptions of the complex needs of older individuals and how health-apps may or may not fit into existing care pathways should also be explored. Essential to this is clarity around what healthcare professionals expect the role of digital health to be among older persons, and whether this differs from what older persons themselves believe the role of digital health may be. Our finding that the gap between willingness to use health-apps, and recommendation of health-apps by healthcare professionals increase with age, suggests that older persons should be asked more frequently, if in fact they would like to use health-apps, as currently, our findings suggest that healthcare professional assumptions play a large role in this process.

Furthermore, this lack of confidence in recommending health-apps to older persons, who are more likely to be multi-morbid or suffer from complex conditions, may also be exacerbated by a lack of awareness of suitable technologies, and concerns regarding the perceived efficacy of health-apps in this age group. Previous research, employing a novel discrete-choice experiment which asked healthcare professionals which of 2000 hypothetical health-apps they would be most likely to prescribe to patients, has shown that healthcare professionals from the UK were significantly more likely to recommend health-apps if evidence of effectiveness was available [17]. This same study found that recommendations from other healthcare professionals and regulatory stamps of approval all increased the likelihood of recommending health-apps to patients. Therefore, any lack of confidence or concerns regarding the perceived efficacy or risks of health-apps, particularly for those dedicated to older persons, could be assuaged using dedicated health-app formularies, such as those provided by ORCHA, the Bundesinstitut für Arzneimittel und Medizinprodukte (BfArM) Digital Health Applications (DiGA) library, and the NHS.

Our findings that both being male or BAME (age being controlled as a factor) led to a statistically significant higher odds of having used a health-app and being recommended a health-app by a HCP is also of particular interest. This may point to problems of equal digital health inclusion among sexes as highlighted by this recent publication exploring the effect of inequities and biases of app design that may dissuade women from engaging with digital health as much as men [29]. The increased odds for BAME respondents may be due to socioeconomic inequalities in the UK, where apps may provide a cheap accessible form of healthcare and are thus used and recommended by HCPs in these areas more frequently to local patients. Furthermore, a recent review by the Race & Health Observatory in the NHS shows widespread ethnic health inequalities in the UK including more distrust in primary care providers [30], the increased odds of using digital healthcare may stem from DHTs being an accessible way to improve health outcomes without having to engage in formal healthcare systems.

Finally, our finding that those living in London were significantly more likely to have used health-apps than those from all other regions of the United Kingdom is also of interest. This finding may be due to the abundance of digital initiatives with London as their central focus, including Digital Health London, Healthy London Partnership and London Procurement Partnership (LPP). Additionally, London is perceived as a key market to digital health developers, with established products including Sleepio and Be Mindful, historically being offered for free within London to promote digital utilisation [31]. These initiatives have almost certainly increased both awareness and acceptability of health-apps disproportionately within the London area, and it is likely that the success of these initiatives played a role in explaining the higher rates of digital acceptance and utilisation, both among members of the public and healthcare professionals, which we observed within this study.

### Strengths/limitations

The main strength of this study is the large sample size which was protected against self-selection of highly digitally engaged individuals through the use of a novel survey-blinding technique. The sample largely represented underlying UK demographics and included respondents from a broad range of locations within the UK, but with the over-recruitment of older individuals. These age groups are typically less involved in digital health research, and therefore, our ability to blind the topic of the survey beforehand, we believe, has enabled greater participation with 776 individuals aged 55 and over taking part in the study, 466 (60%) of which were aged 65 and over. Finally, the survey itself was designed to make the content easy to understand and respond to, a belief which was validated in prior pilot testing of the survey; while a short survey design mitigated the impact of survey fatigue and inaccurate responses, which are typically more common within longer surveys.

This study also has several limitations that should be considered. Firstly, despite blinding prospective respondents to the survey topic, the use of a digital platform to administer the survey may have resulted in an over-representation of those with at least basic knowledge of technology, thereby potentially overestimating the digital literacy and willingness to use health-apps among this group. Future research should aim to provide surveys in other formats, including in-person and telephone to definitively determine if the medium of the survey affected the findings. Secondly, the availability of the survey in the English language only may have also led to a more limited sample as this may be exclusionary to certain BAME populations, especially older BAME generations that may have been first-generation migrants to the UK who may struggle more with reading and writing in the English language. Thirdly, it is important to note the impact of time and the potential for recall bias on the outcomes under consideration. Our primary outcome of willingness to utilise health-apps was measured in real-time, asking respondents whether or not they would use health-apps today. However, secondary outcomes including recommendations to use health-apps by healthcare professionals are, by definition, historical in nature. Therefore, it is possible that this outcome may be confounded by prior historical beliefs regarding the value of health-apps and may not reflect the opinions and belief systems of healthcare professionals in the present day.

In addition, this survey focuses specifically on healthcare apps, further research may be needed to assess the willingness to use and actual use of wearables such as fitness trackers and smartwatches as well as home/environmental sensors and monitors for older populations. Also, our findings that being male or being part of the BAME community leads to significantly higher odds of historical use and being recommended apps by HCPs is of particular interest, while delving further would be outside the aims of this paper, future research may want to pursue this to find the causes behind this difference. Furthermore, while this study touches on the reluctance of healthcare professionals to recommend apps to older people, future research may want to explore this more in-depth also.

## Conclusion

To conclude, this study, utilising a novel survey design, found that while willingness to use health-apps was significantly lower among older persons, overall acceptance was still very high. However, decisions by healthcare providers to offer digital health technologies significantly less to older persons, may result in increasing unmet needs and missed opportunities. These findings suggest that more should be done by healthcare providers to recommend high-quality digital health to older persons who are generally positive about their use, particularly those designed with older persons in mind. Discussions with patients regarding their perceptions of the value and role of digital health are essential to this process. This may in turn result in considerable improvements in healthy and independent ageing.

## Data Availability

The data that support the findings of this study can be made available upon request. Correspondence and requests for materials should be addressed to simon.leigh@orcha.co.uk.
